# Supplementary material for: A Critical View of the Application of the APEX Software (Aqueous Photochemistry of Environmentally-Occurring Xenobiotics) to Predict Photoreaction Kinetics in Surface Freshwaters
Source: Molecules. 2019 Dec 18;25(1):9. doi: 10.3390/molecules25010009 (PMC7017383; doi:10.3390/molecules25010009)
Supplement: Supplementary file 1 [file molecules-25-00009-s001.zip › 3.2.4_gcc-4.4.0.zip!/3.2.4_gcc-4.4.0/3.2.4_gcc-4.4.0/doc/octave/html/interpreter/Quitting-Octave.html]

Quitting Octave - Untitled


Next: Getting Help,
Previous: Invoking Octave from the Command Line,
Up: Getting Started


---

### 2.2 Quitting Octave

— Built-in Function: **exit** (status)  
— Built-in Function: **quit** (status)  
> Exit the current Octave session. If the optional integer value
> status is supplied, pass that value to the operating system as the
> Octave's exit status. The default value is zero.

— Built-in Function: **atexit** (fcn)  
— Built-in Function: **atexit** (fcn, flag)  
> Register a function to be called when Octave exits. For example,
>
> ```
>           function last_words ()
>             disp ("Bye bye");
>           endfunction
>           atexit ("last_words");
> ```
>
> will print the message "Bye bye" when Octave exits.
>
> The additional argument flag will register or unregister
> fcn from the list of functions to be called when Octave
> exits. If flag is true, the function is registered, and if
> flag is false, it is unregistered. For example,
> after registering the function `last_words` above,
>
> ```
>           atexit ("last_words", false);
> ```
>
> will remove the function from the list and Octave will not call
> `last_words` when it exits.
>
> Note that `atexit` only removes the first occurrence of a function
> from the list, so if a function was placed in the list multiple
> times with `atexit`, it must also be removed from the list
> multiple times.
